# Health by Knowledge

**Published:** 1921-03-19

**Authors:** 


### Health by Knowledge.

### WHY SOME EXPERIMENTS BREAK DOWN.

A promising experiment in promoting industrial efficiency and comfort has just broken down through neglect of a very obvious precaution. A firm whose workmen were employed in very dirty processes was impressed with the value of allowing them to go home clean and comfortable, and accordingly bathing arrangements were designed and installed. Unfortunately, however, nobody remembered or knew of the false modesty of the working man, and the men concerned simply took no advantage of the scheme, because the arrangements had been made with no regard to privacy. The moral of the incident is that it is useless to indulge in elaborate schemes which ignore the human factor. These arrangements were probably made by university men accustomed to take exercise and bathe and swim together, and to the sight of their bare bodies. They entirely overlooked the fact that the ordinary working man strips far less often, though their experience in Army medical examinations might have taught them this.

One of the commonly amusing experiences of medical men conducting such examinations was the contrast between the readiness of a gently-nurtured youth of the upper classes to strip as a matter of course, and the clumsy shyness of some burly collier or ironfounder. A great deal of impatience among employers who have endeavoured to introduce better health and sanitary conditions in their works has arisen from just this cause. Scientifically the arrangements have been perfect. But they have neglected the human factor. Some trivial point in them has loomed up all-important in the minds of the men concerned, and, of course, without explaining themselves, or perhaps unable to do so, they have refused to take advantage of schemes which would have been of inestimable value to themselves and to their employer. We are learning the lesson nowadays. We smile at some of the explanations and advice given us by modern traffic managers. Yet they know the difference between sheep and men, and they realise that it is impossible to drive thousands of people, but that once they have got the right idea into their heads they will go right of their own accord.

In the conditions of a great deal of present-day medical work explanations are often impossible and sometimes dangerous from the liability to misunderstanding. But one of the greatest dangers of bureaucracy is the tendency to avoid explanations. It may make the machine run smoothly, but it will not make the human being function as he should, and, after all, every individual has tremendous scope for practising "ca' canny." Members of the medical profession are sometimes accused of entertaining a fear of making their knowledge "cheap" by giving away information, but the point of view is wrong, for the man who is best informed is usually the man most ready to seek advice and help. It should be the desire of every member of the medical profession to have an educated constituency. The more men know of medicine and the methods of the modern medical service, the more they will demand its extension. Professional secrecy is increasingly regarded nowadays as professional mumbo jumbo. The doctor has nothing to lose by practising frankness wherever possible. There are obvious limits to the lengths to which he can go in trusting his patients or securing their co-operation, but in all attempts to secure better health methods and to apply sanitary science the human factor is of the utmost importance. An inferior scheme willingly worked by people who understand it will secure better results than the most perfect system which leaves those whom it concerns indifferent or antipathetic.

